# Efficacy and Safety of Adjunctive Lumateperone 42 mg in Major Depressive Disorder: A Pooled Analysis of Demographic and Clinical Subgroups from 2 Phase 3 Randomized Placebo-Controlled Trials

**DOI:** 10.1192/j.eurpsy.2026.10608

**Published:** 2026-07-31

**Authors:** W. R. Earley, S. Durgam, C. Chen, R. Migliore, J. Riise, S. G. Kornstein, R. S. McIntyre, M. Fava

**Affiliations:** 1 Intra-Cellular Therapies, a Johnson & Johnson Company, Bedminster, United States; 2 Johnson & Johnson, Copenhagen, Denmark; 3 Department of Psychiatry, Virginia Commonwealth University School of Medicine, Richmond, United States; 4 Department of Psychiatry, Department of Pharmacology and Toxicology, University of Toronto, Toronto, Canada; 5 Massachusetts General Hospital and Harvard Medical School, Boston, United States

## Abstract

**Introduction:**

Lumateperone is an FDA-approved antipsychotic to treat schizophrenia and depressive episodes associated with bipolar I or bipolar II disorder. Efficacy and safety of lumateperone adjunctive to antidepressant therapy (ADT) was established in 2 Phase 3, randomized, double-blind, placebo-controlled trials (501, NCT04985942; 502, NCT05061706) in patients with major depressive disorder (MDD) with inadequate ADT response.

**Objectives:**

This pooled analysis of Studies 501/502 evaluated lumateperone 42mg+ADT in demographic and clinical subgroups of patients with MDD.

**Methods:**

Data were pooled from Studies 501/502 which enrolled adults with DSM-5–diagnosed MDD with inadequate response to 1-2 ADT in the current depressive episode (Montgomery-Åsberg Depression Rating Scale [MADRS] Total score ≥24, Clinical Global Impression-Severity [CGI-S] score ≥4). Patients were randomized to 6-week oral lumateperone 42mg+ADT or placebo+ADT. MADRS Total and CGI-S scores were evaluated in the pooled population and patient subgroups by demographic and clinical characteristics. Safety was assessed.

**Results:**

The pooled population comprised 950 patients (lumateperone+ADT, n = 471; placebo+ADT, n = 479). Lumateperone+ADT significantly improved MADRS Total score (least squares mean difference vs placebo [LSMD]=‒4.7; effect size [ES]=‒0.59; *P* < .0001) and CGI-S score (LSMD=‒0.6; ES=‒0.59; *P* < .0001) from baseline to Day 43 vs placebo+ADT.

Lumateperone+ADT significantly improved (*P* < .05) MADRS Total score at Day 43 in all patient subgroups vs placebo+ADT based on age (≤40 years: LSMD=‒4.5; >40 years: LSMD=‒4.9), sex (male: LSMD=‒4.2; female: LSMD=‒5.0), region (US: LSMD=‒4.7; non-US: LSMD=‒4.6), disease severity (baseline MADRS Total score <32: LSMD=‒4.6; baseline score ≥32: LSMD=‒5.0), type of ADT (SSRI: LSMD=‒4.9; SNRI: LSMD=‒3.8), number of ADT failures (1 failure: LSMD=‒4.5; 2 failures: LSMD=‒6.3), race (White: LSMD=‒5.0; non-White: LSMD=‒3.2), and baseline anxious distress (Yes: LSMD=-5.9; No: LSMD=-4.0). Lumateperone+ADT significantly improved (*P*<.01) CGI-S score vs placebo+ADT at Day 43 in all subgroups (age, sex, race, region, baseline disease severity, type of ADT, number of ADT failures, baseline anxious distress).

Lumateperone+ADT was generally well tolerated. Common treatment-emergent AEs (≥5%; twice placebo+ADT) were dizziness, dry mouth, somnolence, nausea, and fatigue. No notable changes occurred in body morphology, prolactin levels, cardiometabolic parameters, or extrapyramidal symptom scales with lumateperone+ADT.

**Conclusions:**

In this pooled analysis, lumateperone 42mg+ADT had robust efficacy vs placebo+ADT in demographic and clinical subgroups and was generally well tolerated, indicating lumateperone as an adjunctive treatment option in patients with MDD with inadequate ADT response.

**Disclosure of Interest:**

W. Earley Employee of: Intra-Cellular Therapies, a Johnson & Johnson Company, S. Durgam Employee of: Intra-Cellular Therapies, a Johnson & Johnson Company, C. Chen Employee of: Intra-Cellular Therapies, a Johnson & Johnson Company, R. Migliore Employee of: Intra-Cellular Therapies, a Johnson & Johnson Company, J. Riise Employee of: Johnson & Johnson, S. Kornstein Grant / Research support from: National Institutes of Health and the National Science Foundation, Consultant of: Lilly, Sage Therapeutics, Reunion Neuroscience, Relmada, AbbVie, Gerbera, and Arrivo Bioventures, R. McIntyre Grant / Research support from: CIHR/GACD/National Natural Science Foundation of China (NSFC) and the Milken Institute, Speakers bureau of: Lundbeck, Janssen, Alkermes, Neumora Therapeutics, Boehringer Ingelheim, Sage, Biogen, Mitsubishi Tanabe, Purdue, Pfizer, Otsuka, Takeda, Neurocrine, Neurawell, Sunovion, Bausch Health, Axsome, Novo Nordisk, Kris Pharma, Sanofi, Eisai, Intra-Cellular Therapies, NewBridge Pharmaceuticals, Viatris, AbbVie, and Atai Life Sciences, M. Fava Shareholder of: Compellis; Neuromity; Psy Therapeutics; Revival Therapeutics; Sensorium Therapeutics, Grant / Research support from: AbbVie; Acadia Pharmaceuticals; Aditum Bio Management Company, LLC; Allergan, Alkermes, Inc.; Altimate Health Corporation; Alto Neuroscience, Inc.; Ancora Bio, Inc.; Angelini S.p.A; Aptinyx; Arbor Pharmaceuticals LLC; Avanir Pharmaceuticals Inc.; Axsome; Benckiser Pharmaceuticals, Inc.; BioClinica, Inc.; Biogen; BioHaven; BioShin Limited; Cambridge Science Corporation; Centrexion Therapeutics Corporation; Cerecor; Cybin IRL Limited; Damona Pharmaceuticals; EmbarkNeuro; Eliem Therapeutics LTD; Gate Neurosciences, Inc.; GenOmind, LLC; Gentelon, LLC; Gerbera Therapeutics, Inc.; Happify; Johnson & Johnson; Lundbeck Inc.; Marinus Pharmaceuticals; Medpace, Inc.; Methylation Sciences, Inc.; Millennium Pharmaceutics, Inc.; Minerva Neurosciences; Neuralstem; Neurocrine Biosciences, Inc.; NeuroRX Inc.; Novaremed; Novartis; Otsuka; Pfizer; Premiere Research International; Praxis Precision Medicines; Protagenic Therapeutics, Inc.; Relmada Therapeutics Inc.; Reckitt; Shenox Pharmaceuticals; Stanley Medical Research Institute (SMRI); Taisho; Takeda; University of Michigan; Vistagen; WinSanTor, Inc.; Xenon Pharmaceuticals, Inc.; National Institute of Drug Abuse (NIDA); National Institutes of Health (NIH); National Institute of Mental Health (NIMH); and PCORI, Consultant of: Massachusetts General Hospital, except for Revival Therapeutics and Sensorium Therapeutics

